# Behavior Change Following Pain Neuroscience Education in Middle Schools: A Public Health Trial

**DOI:** 10.3390/ijerph17124505

**Published:** 2020-06-23

**Authors:** Adriaan Louw, Regina Landrus, Jessie Podolak, Patricia Benz, Jen DeLorenzo, Christine Davis, Alison Rogers, Kathy Cooper, Colleen Louw, Kory Zimney, Emilio J. Puentedura, Merrill R. Landers

**Affiliations:** 1 Evidence in Motion, Story City, IA 50248, USA; adriaan@eimpt.com; 2 Big Stone Therapies, Hendricks, MN 56136, USA; regina.landrus@bigstonetherapies.com; 3 Evidence in Motion Pain Fellowship, San Antonio, TX 78232, USA; jpodolak@eimpt.com; 4 Confluent Health, Siesta Key, FL 34242, USA; patty@physicaltherapist.com; 5 180 Therapy and Wellness, Alexandria, VA 22314, USA; Jen@DeLorenzoPT.com; 6 St. Croix Regional Medical Center, St Croix Falls, WI 54024, USA; christine.davis@scrmc.org; 7 SERC Physical Therapy, Webb City, MO 64870, USA; arogers@serctherapy.com; 8 Physical Therapy of Concordia, Concordia, MO 64020, USA; kcooperptc@gmail.com; 9 Ortho Spine and Pain Clinic, Story City, IA 50248, USA; Colleen@eimpt.com; 10 Department of Physical Therapy, School of Health Sciences, University of South Dakota, Vermillion, SD 57069, USA; kory.zimney@usd.edu; 11 Doctor of Physical Therapy Program, Robbins College of Health and Human Sciences, Baylor University, Waco, TX 76798-7303, USA; 12 Department of Physical Therapy, School of Allied Health Sciences, University of Nevada Las Vegas, Las Vegas, NV 89154-3029, USA; merrill.landers@unlv.edu

**Keywords:** pain, neuroscience, education, school, children, behavior change

## Abstract

Chronic pain and the opioid epidemic need early, upstream interventions to aim at meaningful downstream behavioral changes. A recent pain neuroscience education (PNE) program was developed and tested for middle-school students to increase pain knowledge and promote healthier beliefs regarding pain. In this study, 668 seventh-grade middle-school students either received a PNE lecture (*n* = 220); usual curriculum school pain education (UC) (*n* = 198) or PNE followed by two booster (PNEBoost) sessions (*n* = 250). Prior to, immediately after and at six-month follow-up, pain knowledge and fear of physical activity was measured. Six months after the initial intervention school, physical education, recess and sports attendance/participation as well as healthcare choices for pain (doctor visits, rehabilitation visits and pain medication use) were measured. Students receiving PNEBoost used 30.6% less pain medication in the last 6 months compared to UC (*p* = 0.024). PNEBoost was superior to PNE for rehabilitation visits in students experiencing pain (*p* = 0.01) and UC for attending school in students who have experienced pain > 3 months (*p* = 0.004). In conclusion, PNEBoost yielded more positive behavioral results in middle school children at six-month follow-up than PNE and UC, including significant reduction in pain medication use.

## 1. Introduction

The global pain epidemic is well-documented. For example, in the United States (US), it is currently reported that 25.3 million adults suffer from daily chronic pain and it is estimated that 126.1 million adults in the US experience some pain over a three-month reporting period [1,2]. Children and adolescents also struggle with persistent pain with various studies reporting approximately one in six experiencing persistent pain [3,4,5]. In the US, the resulting opioid epidemic has also reached a critical point. It is reported that in 2012, US healthcare providers wrote 259 million prescriptions for opioid pain medications [6] with the US consuming 80% of the global opioid supply, and 99% of the global hydrocodone supply [7]. These staggering numbers, including US daily opioid-related deaths exceeding 130, warrant urgent and extensive attention.

The exact reason for the pain and opioid epidemic is multi-factorial, complicated, and political [3,8]. To address this epidemic, much attention is being given to the current chronic pain and opioid-using sufferer [9,10]. There must, however, be a comprehensive, national, and global initiative to prevent this epidemic. One such initiative may be to teach children more about the neurophysiology and neurobiology of pain, referred to as pain neuroscience education (PNE) [8,11,12]. In clinical practice, PNE is an educational strategy used by healthcare providers that focuses on teaching people in pain about the biological and physiological processes involved in their pain experience [13,14,15]. Current best-evidence provides strong support for PNE used in conjunction with exercise and other healthy lifestyle behaviors to positively influence pain ratings, dysfunction, fear-avoidance, pain catastrophization, limitations in movement, pain knowledge and healthcare utilization [12,16]. PNE researchers have argued that current biomedical models, which are very prevalent in medicine, may be a substantial factor behind the pain epidemic [17,18,19]. In the biomedical model, the health of a person’s tissues is powerfully connected to their pain experience, yet it is well documented that the health of tissues and pain do not necessarily correlate [20,21,22]. As long as patients, healthcare providers, and the general population connect the health of tissues to how much pain someone will experience, it can increase fear-avoidance and pain catastrophization. These beliefs have been shown to be powerful predictors of persistent pain, including use of opioids [23,24].

In line with this reasoning, a series of PNE studies have been designed and conducted for middle school students in the US [8,11,25]. In this approach, it is reasoned that if children are taught healthier beliefs and attitudes about pain and its treatment options, it may in fact have downstream effects later in life [8]. A series of studies ensued to build, test and validate the program, before testing the long-term behavioral changes. In the first study, a PNE curriculum was built and tested on 147 middle-school students in regard to their knowledge of pain as well as attitudes and beliefs regarding pain. In this simple pre-, post-PNE study, knowledge of pain shifted significantly, in line with clinical studies as well as fostering healthier attitudes and beliefs regarding pain, especially chronic pain [8]. In a second follow-up study (Benz, et al.—submitted for publication), PNE delivered to middle-school students was able to powerfully influence fear-avoidance of physical activity in the presence of pain. In terms of delivery, Podolak et al. [25], showed that an animated, video-delivery of the same PNE content yielded similar results to live, in-person, physical therapist-delivered PNE, thus showcasing an ability to scale such a PNE middle school project. More recently, Louw et al. [11], compared the newly-designed PNE middle school program to current content taught to middle school children regarding pain. The results supported current clinical studies whereby PNE-delivered content reduced fear, while current biomedically driven middle-school content increase fear and fear-avoidance [12]. Additionally, as expected, the PNE program yielded superior results in terms of pain knowledge compared to current school curricula.

However, an important question remains unanswered. Will a program such as this yield any downstream positive behavioral results? In all mass-education, population-based studies, behavior change is the key to success [26]. For example, in smoking-cessation programs, the ultimate result is smoking cessation [27]. To truly determine if such a PNE program results in behavior change would need to be demonstrated. It is important to realize that education typically as a stand-alone intervention for behavior change is very limited. For example, the US spends $2.1 trillion annually on smoking-cessation programs and yields a 20–25% success rate [27,28,29]. With the development and validation of the PNE middle school program complete, this study set out to examine if PNE delivered to middle-school students would yield positive behavior changes during/after the school year, as measured six-months later. In line with previous PNE studies, three educational approaches were established for testing: one-time, in-person PNE delivered session (PNE); one-time, in-person educational session using current school or ‘usual curriculum’ content on pain (UC) and PNE session followed by two booster sessions (PNEBoost). Three aims were formulated. Aim One was to determine if there would be any difference between the three groups, six months later in terms of school, physical education (PE), sports and recess attendance/participation as well as healthcare choices (doctors’ visits, rehabilitation visits and/or pain medication use). The second aim was to compare the three groups’ six-month outcomes taking into consideration demographic variables (gender, pain at the time of the intervention, having experienced pain >3 months, knowing someone with chronic pain, high level of fear-avoidance and pain knowledge). The third and final aim was to determine which demographic variables were most associated with school attendance, PE participation, recess participation, and sports participation in the ensuing six months.

## 2. Materials and Methods

### 2.1. Participants and Recruitment

**Figure 1:**
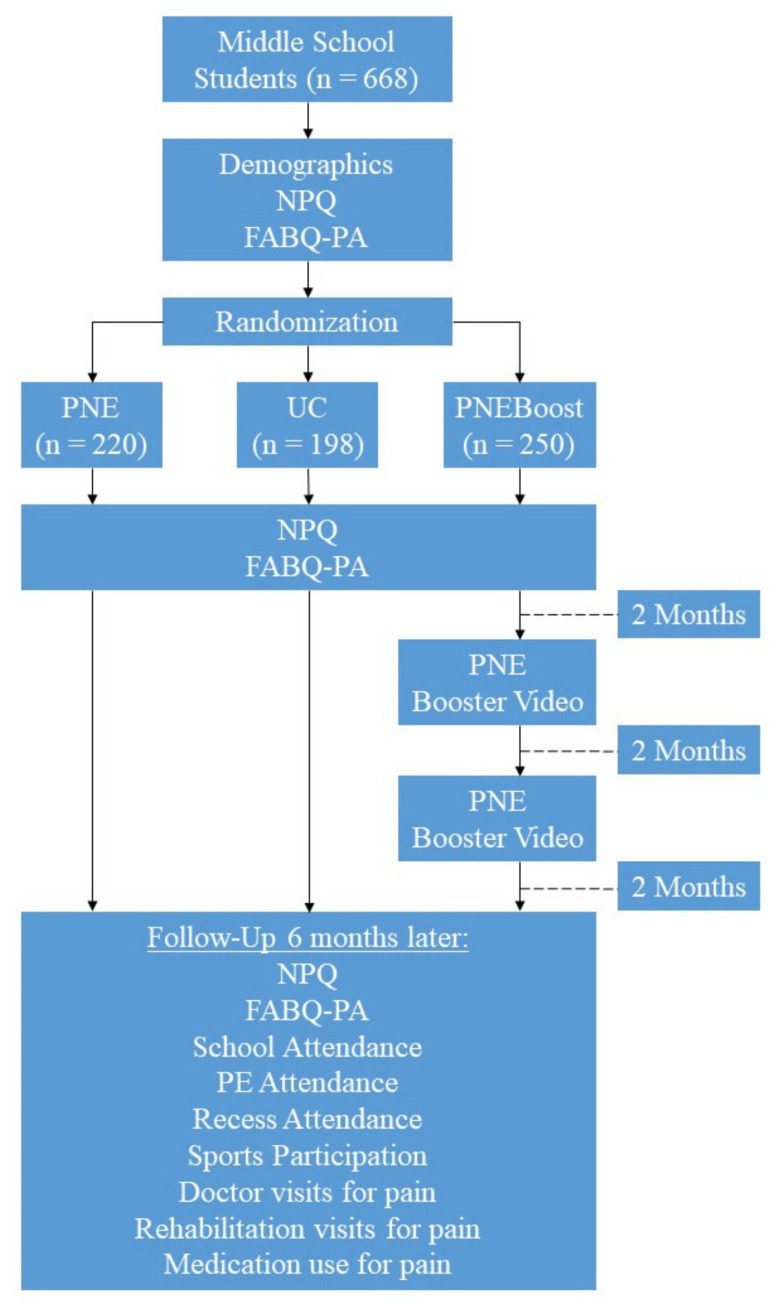
Study Flow Diagram.

In previous middle school PNE studies [8,11,25], it has been shown that 6th and 7th grade school children (aged 11 to 13) are better equipped to take on more complex thoughts, and therefore, more likely to benefit from education about pain. We chose to recruit 7th graders in public and private schools in various states in the US because of this and also because they would be more likely to participate in more physical activities and sustain more sporting or recreational injuries than their younger counterparts [2,11,25]. For the purpose of this study, clinicians who were familiar with PNE, met minimum training requirements and who had access to middle schools were recruited similar to previous middle school PNE studies [8,11,25]. Written consent from the schools for participation in this study was obtained by each clinician participating in the study. In all, 6 clinicians met the criteria for delivering the PNE, and obtaining consent from the schools. Each school was provided with the goals of the educational programming, layout, and necessary examples of the proposed lecture and outcome measures. Since each school had a different approval process (private, public, different states, etc.), it was left up to each school’s administration to determine its own internal procedure for approval, varying between school board approvals, superintendent approvals and/or individualized parental consent. Consent forms were collected from each location prior to the start of the study. All students in the studies had the option to opt out of the study. No personal identifiable data was collected. Once each school consented, teachers were instructed and familiarized with the intended study and the one-time 45-min session was entered into the class schedule, with the exception of the PNEBoost group, which scheduled 2 additional booster sessions (see intervention section). Classes were randomly assigned per concealed envelope system to either receive a PNE lecture or usual care (UC) or PNE plus booster sessions (PNEBoost) (Figure 1). Some clinicians were only able to secure one class/location, while others delivered education at multiple sites. The only exclusions set for the study were students who did not want to attend the class, parents objecting to their child attending the class, or students not proficient in writing and reading English. The research project was conducted according to the Declaration of Helsinki and was approved by the Institutional Review Board for research involving human subjects (University of South Dakota – IRB-19-149).

### 2.2. Intervention

#### 2.2.1. Pain Neuroscience Education (PNE)

The content of middle-school PNE program is well documented [8,11,25]. Given the short duration of the class period, an abbreviated 30-min, 32-slide PowerPoint™ (Microsoft Corporation, Redmond, WA, USA) presentation was developed, allowing ample time for survey completion prior to and following the PNE lecture. The PowerPoint™ presentation’s main themes included a discussion of peripheral sensitization, central sensitization, and biopsychosocial factors associated with pain, threat appraisal of the brain, nociception, stress and endocrine responses in pain as well as various therapeutic endogenous strategies to ease pain [30,31,32,33]. Various images, metaphors and examples were used to convey the PNE to the students [34]. Following the formal presentation by the attending clinician and completion of the immediate post-PNE surveys, participants were encouraged to ask questions. The presentation did not specifically address or target any questions contained in the outcome measures.

#### 2.2.2. Pain Neuroscience Education Plus Booster (PNEBoost)

The second group of students (PNEBoost) received the exact same intervention and procedures as the PNE group, except 2 and 4 months later, a booster session was provided for the students via video-delivery (Figure 1). A previously tested video-presentation of the PNE was broken into 2 parts (approximately 10 min each) and shown at the two designated time-periods by the teachers as a means to provide a refresher of the content.

#### 2.2.3. Usual Care (UC)

Teaching students their current content pertaining to biology, pain and injuries constituted usual care. The content has been established and tested in a previous PNE middle school study [11]. The UC contained descriptions of acute and chronic pain, chronic injuries, acute injuries, soft (muscle, tendon) injuries and hard injuries (bones, fractures), cuts, sprains and strains, fractures, concussions as well as dehydration and hypothermia. In essence, the UC constituted a biomedical sports medicine presentation. Care was taken to ensure the exact verbiage of the curricula were intact to represent UC that current 7th grade students receive in the US. The UC presentation was 34 color PowerPoint™ slides, similar in duration as PNE and with images from the current curricula.

### 2.3. Outcome Measures

Prior to formal outcome measures, students completed a demographic section capturing their age, gender, grade, and participation in sports. Additionally, the demographic survey also enquired about various personal aspects pertaining to pain including: (a) currently experiencing pain; (b) pain rating (numeric pain rating scale [NPRS]); (c) past experiences about pain; and (d) family with persistent pain. No personally identifiable information was captured, and pages were coded to allow matching pre-, immediate post- and 6-month follow-up surveys. Two outcome measures were used for the student to examine their knowledge of pain and fear of physical activity in the presence of pain.

#### 2.3.1. Pain Knowledge

Pain knowledge was measured using the revised neurophysiology of pain questionnaire (rNPQ). The original NPQ is a 19-point questionnaire requesting ‘true’; ‘false’; or ‘not sure’ answers to statements, with higher scores indicating more correct answers. Since the development of the NPQ a statistical analysis of the NPQ has led to the development of a revised NPQ with 12 questions which removed ambiguous questions [35]. The revised 12-question rNPQ was used in this study. The questionnaire was adapted similar to a previous study to make it easier for students to understand, e.g., “nociception” was replaced with “danger messages” [30]. No information is currently available on what constitutes a meaningful shift in rNPQ score. The mean increase in rNPQ for PNE middle school studies using the current curriculum is 27.4% (range 23.6–31.3%) [8,11,25].

#### 2.3.2. Fear Avoidance of Physical Activity

To assess the student’s fear of physical activity, the physical activity sub-scale of the fear-avoidance beliefs questionnaire (FABQ) was used. The FABQ is a 16-item questionnaire that was designed to quantify fear and avoidance beliefs in individuals with low back pain. The FABQ has two subscales: (1) a 4-item Physical Activity (PA) scale to measure fear avoidance beliefs about physical activity, and (2) a 7-item Work (W) scale to measure fear-avoidance beliefs about work. Given this study was administered to school students without low back pain, only the FABQ-PA was used. Each item is scored from 0 to 6 with possible scores ranging between 0 and 24 for the physical activity subscale, with higher scores representing an increase in fear-avoidance beliefs. The FABQ has demonstrated acceptable levels of reliability and validity in previous studies [36,37,38]. Presence of avoidance behavior is associated with increased risk of prolonged disability and work loss. It is proposed that FABQ-PA >14 is associated with a higher likelihood of not returning to work [39,40], and a score >15/24 is classified as a “high score” [41]. Given that the FABQ was developed for people in pain, the wording was altered to reflect their agreement to physical activity in the event they “were to experience pain”, versus “currently experiencing pain”. This methodology has been used in previous PNE middle school studies [11,25].

#### 2.3.3. Behaviors

In line with the aims of the study, a survey was developed by the researchers to assess various behavioral changes 6 months after the initial intervention (end of the school year). The survey asked students:

- Did they miss school in the last 6 months due to pain—yes/no; if yes, how many days?
- Did they miss PE in the last 6 months due to pain—yes/no; if yes, how many days?
- Did they miss recess in the last 6 months due to pain—yes/no; if yes, how many days?
- Did they miss participation in sports in the last 6 months due to pain—yes/no; if yes, how many days?
- Did they see a doctor in the last 6 months due to pain—yes/no; if yes, how many times?
- Did they attend rehabilitation (physical therapy, chiropractic, occupational therapy and/or massage therapy) in the last 6 months due to pain—yes/no; if yes, how many times?
- Did they take any pain medication in the last 6 months due to pain—yes/no?

The rNPQ and FABQ-PA were administered before, immediately after the PNE, UC and PNEBoost lecture and at 6-month follow-up (Figure 1). Six months after the initial intervention, teachers with guidance from the attending clinicians asked students to complete the behavior change survey. To avoid influencing answers to the outcome measures, any questions that arose during the completion of these forms were addressed by the attending teachers and not the presenters of the PNE or UC. Upon completion, the surveys were placed into envelopes, sealed and sent to an independent research assistant who was blinded to group allocation and who entered the data into an Excel document for analysis.

### 2.4. Statistical Analysis

Data were analyzed using SPSS version 24.0 (IBM SPSS Statistics for Windows, Armonk, NY, USA) and α = 0.05.

#### 2.4.1. Aim 1: 6-Month Outcomes

To determine if there was a difference among the 3 groups (PNE, PNEBoost, and UC), non-parametric Kruskal–Wallis tests were used to analyze each of the following outcome variables—school attendance (days missed), PE attendance (days missed), participation in recess (days missed), and participation in sports (days missed). A non-parametric approach was used to decrease the influence of outliers. Since there were no outliers for pain knowledge (rNPQ score) and fear of physical activity (FABQ-PA score), those two outcomes were analyzed using one-way ANOVAs. For the 6-month outcome variables that were categorical (i.e., taking medication for pain (yes or no), seeing a doctor for pain (yes or no), and attending rehabilitation for pain (yes or no)), the proportions among the 3 groups was analyzed using chi square analyses.

#### 2.4.2. Aim 2: Interactions

Interactions among group by demographic variables were analyzed using 3 (group: PNE, PNEBoost, and UC) X 2 (detailed next) ANOVAs for each of the 6-month outcomes. The following demographic variables were analyzed for an interaction with group: gender (boy or girl), pain at time of intervention (yes or no), chronic pain (>3 months) at time of intervention (yes or no), knowing someone with chronic pain (yes or no), fear of physical activity at time of intervention (>15 or ≤15 points on the FABQ-PA), and pain knowledge change from intervention (>2 or ≤2 points improvement on the rNPQ). To decrease the influence of outliers, outliers with z scores beyond +/− 3.0 were omitted from the analyses.

#### 2.4.3. Aim 3: Prediction

Linear multiple regression was used to determine which of the following variables were most associated with school attendance—PE participation, recess participation, and sports participation in the ensuing 6 months: gender, duration of pain at time of intervention, knowing someone with chronic pain, NPRS at time of intervention, FABQ-PA change score, and rNPQ change score.

## 3. Results

### 3.1. Participants

During the course of the study, 668 middle-school students received one of the designated interventions and were followed for 6 months post-initial intervention (Table 1; Figure 1). In all, 16 schools in six different states (Kentucky, Minnesota, Missouri, South Dakota, Virginia and Wisconsin) participated in the study, delivered by six clinicians (five physical therapists and one occupational therapist).

### 3.2. Aim 1: 6-Month Outcomes

There were no differences among the 3 groups (PNE, PNEBoost, and UC) for any of the following 6 month outcome variables except for rNPQ score (*p* = 0.023): school attendance (*p* = 0.949), PE participation (*p* = 0.909), recess participation (*p* = 0.671), sports participation (*p* = 0.779), and FABQ-PA (*p* = 0.871). Descriptive statistics for these analyses are in Table 2.

**Figure 2:**
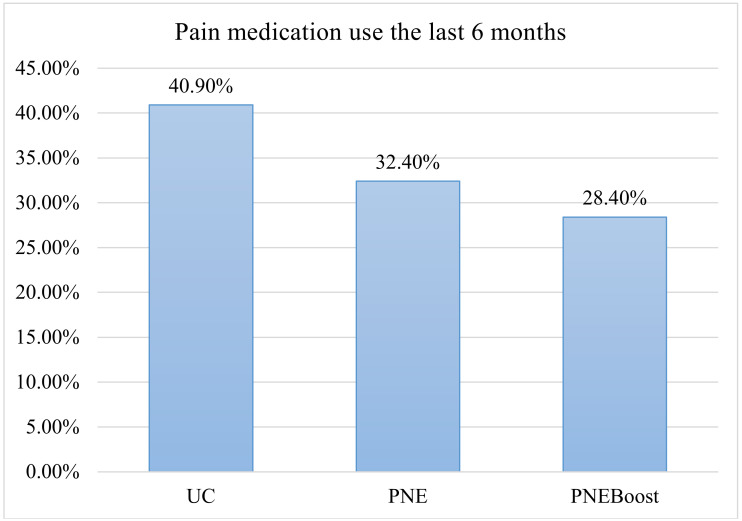
Self-reported use of pain medication in the 6-month period following initial intervention.

Tukey post hoc analysis revealed that PNEBoost had higher scores on the rNPQ at 6 months compared to the UC group (*p* = 0.017). There were no other statistically significant pairwise comparisons (*p* > 0.310). There were no differences in the proportions of participants among the 3 groups for those seeing a doctor for pain (*p* = 0.585) and attending rehabilitation for pain (*p* = 0.972); however, there was a significant difference among the 3 groups in the proportion of participants (PNE = 32.4%; PNEBoost = 28.4%; UC = 40.9%) who took medication for pain in the 6 month period following the intervention (*p* = 0.024). The absolute difference in proportions between the UC and the PNEboost groups was 12.5% (UC-PNEboost); however, the relative difference in the proportions was 30.6% (UC-PNEboost/UC) (Figure 2).

### 3.3. Aim 2: Interactions

All *p* values for the interactions and main effects from the analyses below are located in Table 3. 

Descriptive statistics for Aim Two can be found in the Appendix.

Statistically significant results for each analysis are detailed below.

*Group by gender*. There were no group by gender interactions for any of the 8 outcome variables at 6 months; however, there was a gender main effect for PE participation with girls missing more days (mean days = 1.2, 95% CI: 0.9 to 1.4) than boys (mean days = 0.5, 95% CI: 0.3 to 0.8) regardless of group membership. There was another gender main effect for FABQ with girls exhibiting more fear avoidance beliefs (FABQ = 15.1, 95% CI: 14.5 to 15.6) than boys (FABQ = 13.5, 95% CI: 13.0 to 14.0) regardless of group membership. Lastly, there was a group main effect for rNPQ with those in the PNEBoost group exhibiting higher pain knowledge (rNPQ = 5.1, 95% CI: 4.8 to 5.4) compared to those in the UC group (rNPQ = 4.5, 95% CI: 4.2 to 4.8), *p* = 0.025. There were no other pairwise differences for that main effect.

*Group by pain at intervention*. The only interaction for the group by pain at intervention analysis was for the number of rehabilitation visits for pain (*p* = 0.010). To break down this interaction, two one-way ANOVAs were run, one for those who did not have pain at the intervention (*p* = 0.544) and one for those who did (*p* = 0.028). Post hoc pairwise analyses revealed that the PNEBoost group had significantly fewer visits to rehabilitation (mean visits = 0.4, 95% CI: 0.2 to 0.6) compared to the PE group (mean visits = 1.2, 95% CI: 0.7 to 1.8). There were no group main effects except for the rNPQ outcome (*p* = 0.025) with pairwise comparisons revealing that the PNEBoost had significantly more pain knowledge at 6 months (rNPQ = 5.2, 95% CI: 4.9 to 5.5) compared to the UC group (rNPQ = 4.5, 95% CI: 4.1 to 4.9), *p* = 0.020. There were 3 statistically significant main effects for pain at intervention: school attendance (*p* = 0.005), doctor visits for pain (*p* = 0.013), and rNPQ score (*p* = 0.025). Those with pain at the intervention missed more days of school (missed days = 0.7, 95% CI: 0.5 to 0.8) than those without pain (missed days = 0.4, 95% CI: 0.3 to 0.5) regardless of group membership. Those with pain at the intervention also had more visits to the doctor for pain (visits = 1.5, 95% CI: 1.2 to 1.8) compared to those without pain (visits = 1.1, 95% CI: 0.9 to 1.3) regardless of group membership.

*Group by chronic pain at intervention.* The only statistically significant interaction was for school attendance (*p* = 0.004). To break down this interaction, two one-way ANOVAs were run, one for those who did not have chronic pain at the intervention (*p* = 0.583) and one for those who did (*p* = 0.046). Those with chronic pain in the PNE group missed less days of school (mean days = 0.6, 95% CI: 0.2 to 1.0) than the UC group (mean days = 1.6, 95% CI: 0.5 to 2.7), *p* = 0.037. There were no other statistically significant pairwise differences for those who had chronic pain at the intervention. The only statistically significant group main effect was for PE participation (*p* = 0.039). The only significant pairwise comparison demonstrated that the PNEBoost group missed more days of PE (missed days = 1.7, 95% CI: 1.3 to 2.1) compared to the PE group (missed days = 0.9, 95% CI: 0.5 to 1.3), *p* = 0.034, regardless of the presence of chronic pain at the intervention. There were several statistically significant main effects for chronic pain at intervention (Table 2). Those with chronic pain missed more days of PE (chronic pain = 1.8, 95% CI: 1.3 to 2.4; no chronic pain = 0.7, 95% CI: 0.5 to 0.9) and sports participation (chronic pain = 1.8, 95% CI: 1.3 to 2.3; no chronic pain = 0.9, 95% CI: 0.7 to 1.0). In addition, they had more doctor visits for pain (chronic pain = 2.3, 95% CI: 1.9 to 2.7; no chronic pain = 1.1, 95% CI: 0.9 to 1.3) and more rehabilitation visits for pain (chronic pain = 1.1, 95% CI: 0.7 to 1.5; no chronic pain = 0.5, 95% CI: 0.4 to 0.7). Lastly, those with chronic pain also had lower rNPQ scores at the end of 6 months (chronic pain = 4.6, 95% CI: 4.1 to 5.1; no chronic pain = 4.8, 95% CI: 4.7 to 5.0).

*Group by knowing someone with chronic pain.* Of the 8 outcomes, there were two statistically significant groups by knowing someone with chronic pain interactions, rNPQ (*p* = 0.010) and FABQ-PA (*p* < 0.001). To break down the interaction for rNPQ, two one-way ANOVAs were run, one for those who did not know someone with chronic pain (*p* = 0.317) and one for those who did know someone (*p* = 0.001). For those who did know someone, both the PNEBoost group (rNPQ = 5.2, 95% CI: 4.8 to 5.5) and the PNE group (rNPQ = 4.8, 95% CI: 4.5 to 5.2) had better pain knowledge than the UC group (rNPQ = 4.2, 95% CI: 3.8 to 4.6), *p* < 0.001 and *p* = 0.041, respectively. There were no other statistically significant pairwise differences. For those in the UC group, knowing someone meant lower pain knowledge (rNPQ = 4.2, 95% CI: 3.8 to 4.6) than those who did not know someone (rNPQ = 5.6, 95% CI: 4.6 to 6.5), *p* = 0.009. To break down the interaction for FABQ-PA, two one-way ANOVAs were also run, one for those who did not know someone with chronic pain (*p* = 0.009) and one for those who did know someone (*p* = 0.269). For those not knowing someone with chronic pain, those in the UC group had more fear avoidance beliefs (FABQ-PA = 15.6, 95% CI: 13.9 to 17.4) than the PNE group (FABQ-PA = 12.1, 95% CI: 10.7 to 13.5), *p* = 0.002. There were no other pairwise differences. Knowing someone with chronic pain had a statistically significant effect on missed days of school (knowing someone = 0.6, 95% CI: 0.5 to 0.7; not knowing someone = 0.3, 95% CI: 0.1 to 0.4), missed days of PE participation (knowing someone = 1.0, 95% CI: 0.8 to 1.2; not knowing someone = 0.5, 95% CI: 0.2 to 0.9), and missed days of sports participation (knowing someone = 1.1, 95% CI: 0.9 to 1.3; not knowing someone = 0.6, 95% CI: 0.3 to 0.9). Knowing someone with chronic pain also meant more visits to the doctors for pain (knowing someone = 1.4, 95% CI: 1.2 to 1.6; not knowing someone = 0.9, 95% CI: 0.6 to 1.2) and more visits to rehabilitation for pain (knowing someone = 0.7, 95% CI: 0.6 to 0.9; not knowing someone = 0.4, 95% CI: 0.1 to 0.7).

*Group by fear of physical activity at time of intervention.* There were no statistically significant interactions between group and fear of physical activity at time of intervention on any of the 8 outcome variables. However, there was a statistically significant group main effect for rNPQ with the PNEBoost group having significantly better pain knowledge (rNPQ = 5.1, 95% CI: 4.8 to 5.4) compared to the UC group (rNPQ = 4.5, 95% CI: 4.1 to 4.8), *p* = 0.013. Those with high fear of physical activity missed more days of PE (mean days = 1.3, 95% CI: 1.0 to 1.6) than those with low fear of physical activity (mean days = 0.6, 95% CI: 0.4 to 0.9). Those with high fear of physical activity also missed more days of sports participation (mean days = 1.4, 95% CI: 1.1 to 1.6) compared to those with low fear of physical activity (mean days = 0.8, 95% CI: 0.6 to 1.0). Those with high fear of physical activity at the intervention also had more fear of physical activity at the 6-month point (FABQ-PA = 16.3, 95% CI: 15.7 to 16.9) than those with low fear of physical activity (FABQ-PA = 13.1, 95% CI: 12.7 to 13.6).

*Group by pain knowledge change from intervention.* There were two statistically significant interactions between group and pain knowledge change for school attendance (*p* = 0.023) and rNPQ (*p* = 0.049). To break down the school attendance interaction, two one-way ANOVAs were run, one for those whose pain knowledge improved (*p* = 0.122) and one for those whose pain knowledge did not (*p* = 0.167). Since neither were statistically significant, 3 t-tests for the comparison of those who improved and those who did not for each of the three intervention groups were conducted and revealed only one significant difference for the PNEBoost group. Specifically, those participants who improved due to the PNEBoost intervention missed less days of school (mean days = 0.4, 95% CI: 0.2 to 0.6) compared to students who did not improve (mean days = 0.8, 95% CI: 0.4 to 1.1), *p* = 0.033. For the rNPQ interaction, two one-way ANOVAs were also run, one for those one for those whose pain knowledge improved (*p* = 0.027) and one for those whose pain knowledge did not (*p* = 0.137). For those whose pain knowledge improved, the PNEBoost group had higher rNPQ scores (mean = 5.4, 95% CI: 5.0 to 5.7) compared to the PNE group (mean = 4.6, 95% CI: 4.3 to 5.1), *p* = 0.025. There were no other pairwise differences. There was a statistically significant main effect for rNPQ improvement with those who improved having fewer missed recesses (mean days = 0.5, 95% CI: 0.4 to 0.6) than those whose pain knowledge did not improve (mean days = 0.2, 95% CI: 0.1 to 0.4), *p* = 0.004, regardless of group membership.

### 3.4. Aim 3: Prediction

Detailed results for each of the regression analyses are in Table 4. Summary of each regression analysis are broken down below by dependent variable.

*School attendance.* Three variables (duration, rNPQ improvement, and knowing someone with chronic pain) were associated with school attendance (*p* < 0.001) (Table 5); however, the variance explained was only 6.4% (R^2^ = 0.64). These results suggest that longer duration of pain at the intervention, knowing someone with chronic pain, and improved score on pain knowledge before and after the intervention were associated with more days missed from school in the 6 months following the interventions.

*PE participation.* Three variables (gender, duration of pain, and FABQ change) were significantly associated with PE participation (*p* < 0.001) and explained 6.8% of the variance in PE participation (Table 5). These results suggest that being a girl, having longer duration of pain, and having more fear avoidance beliefs were associated with more missed days from PE at school in the subsequent 6 months from the interventions.

*Sports participation.* Only one variable, duration of pain, was associated with sports participation (*p* < 0.001) (Table 5). This variable explained 5.9% of the variance in sports participation and suggests that the longer the duration of pain at the intervention the more likely one would miss participating in sports in the subsequent 6 months.

*Recess participation.* Two variables, duration and rNPQ improvement, were both significantly associated with recess participation (*p* = 0.003) (Table 5); however, these variables only explained 2.7% of the variance in recess participation. Again, the longer the duration of pain and more knowledge improvement were both associated with more missed recesses in the 6 months after the intervention.

## 4. Discussion

This is the first public health trial using PNE in middle schools with long-term follow-up to assess behavior change. A single PNE session with subsequent booster sessions (PNEBoost) results in significant less use of pain medication during the school year for middle school students. Additionally, taking demographic variables into consideration, PNEBoost yielded superior results in regard to school attendance and attending fewer rehabilitation visits for pain, compared to PNE and UC. Students having experienced pain more than three months were significantly more likely to miss school, recess, PE and sports participation six-months later.

The biggest positive finding of the study is that the students in the PNEBoost group used significantly less pain medication in the subsequent school year compared to the UC group. This result solidifies the argument that upstream efforts for downstream effects, especially for medication use, is an area that needs more attention [8]. Even though the type of medication was not studied and not specific to opioids, early exposure to pain medication, including non-opioids, has been cited as a major concern for later use, misuse and addiction to opioid pain medication [42,43,44,45]. It is also imperative to understand that non-opioid medication, i.e., anti-inflammatory medication, has significant risk by itself, thus highlighting again the positive result from this study [46,47]. From a behavioral perspective, the type of medication use is also not as important as the behavior tying use of pain medication to a pain experience. Over time, habitual, repetitive use of pain medication (regardless the type) when someone experiences pain, may develop into a habit, which may have devastating consequences long-term. In this study, the PNEBoost students used less pain medication and even though its true long-term effect (years later) was not studied, it warrants additional and future research with true long-term follow-up.

In addition, of note in this study is the fact that one-third of the students at the time of the intervention were experiencing pain and one-in-seven had experienced pain more than 3 months. Pain is not isolated to adults, but is common in middle school students and may drive their choice of treatments, including use of pain medication [3,4,5]. An interesting observation is that many behaviors related to pain in adolescents are often driven by parents and caregivers [48,49]. In this study, there was no difference in general between the groups for school attendance, PE attendance, recess attendance, sports participation, doctor’s visits and rehabilitation visits, but there was for use of pain medication. It could be argued that parents are intricately involved with decisions and actions associated with attending school, sports and medical appointments, while teachers may guide PE and recess participation, which may explain why no difference was observed. The question then arises why pain medication use was different, especially since the PNEBoost and UC group had similar exposure to family members with chronic pain. Future studies exploring the influence of parents and caregivers in decision-making of pain medication-use in adolescents are needed to further examine this phenomenon.

The students attending the various sessions presented with various demographic variables that may have impacted, positively and negatively, their learning experience for the three educational approaches. These included gender, currently experiencing pain, having experienced pain more than three months themselves, living with/knowing someone with chronic pain, high levels of fear of physical activity and pain knowledge. These variables underscore individual human nature, including the individual pain experience that is unique to every human being [18,50]. In general, the PNEBoost group responded more favorably to the educational session when taking these variables into consideration. For example, students who attended the PNEBoost session while experiencing pain had significantly fewer visits to rehabilitation in the next six months compared to the PNE group. Students who had experienced pain for more than three months that attended the PNEBoost session missed less school than similar students in the UC group. The results also show that PNEBoost students whose pain knowledge improved missed less days at school than PNEBoost students whose pain knowledge did not improve. These results are important, as they imply that improved pain knowledge may be a key element to drive behavior change, concurring with preliminary work done in this area [8,11,25].

An intriguing aspect of this study is that in all of the results, PNEBoost seem to be superior to a single-stand-alone PNE session. This finding is in line with current learning theory of repeat exposure and booster sessions to solidify key messages during educational exposure [26,51]. Behavior change is challenging and repeat exposure is a key element [26]. These results would imply that a curriculum with repeat procedures, interactive tasks, teaching guides, etc., are needed to truly impact change. In clinical practice treating patients with chronic pain, it is uncommon to have immediate, significant changes after one treatment session using PNE [52,53,54]. This sample was not a pain-free population, but resembled current statistics showing children in school have high rates of pain, experience pain for quite some time, and live with/around people with pain. It would thus make sense that, just like in clinical practice, a more robust program (PNEBoost) is needed to change behavior than a single-session PNE. Even more intriguing is the notion of building a pain program for children in school suffering from pain, again to potentially influence downstream choices into adulthood [8].

The main goals of this study were to examine the efficacy of three different educational models. The results, however, also yielded valuable “pain” insight into the life of a middle school student, which can/should be used to build future programs to continue this line of study. Past experiences powerfully influence future experiences and, in this study, having experienced pain more than 3 months powerfully predicted school attendance, PE attendance, recess attendance and sports participation [24,55]. Various studies have shown that school, PE, recess and sports participation/attendance have significant influences on various outcomes including drug use, teen-pregnancy, substance abuse, etc., down the road [7,56]. The results of this study show that various factors such as having experienced pain, pain knowledge, knowing someone in pain and fear-avoidance predict school, PE, recess and sports participation/attendance. These factors, in turn, are positively influenced by PNE and PNEBoost, again substantiating the fact that a dedicated pain program/curriculum must be explored in schools. This concurs with the fact that students having experienced chronic pain had lower rNPQ scores at the end of the six-month follow-up, compared to students having not experienced pain more than three months. Parents and caregivers powerfully influence decisions made by children, including health-related choices [5,48,49]. In this study, three out of four children knew/lived with/around someone with chronic pain, which may instill certain care-seeking behaviors related to pain, including medication use.

### Limitations

This study contains various limitations. The sampling was not a true randomization, but rather a convenience randomization of groups of students versus individual students. This results in a heterogenous sample between the three groups, i.e., UC group experienced less pain at the time of the intervention than PNE and PNEBoost. The duration of each intervention was the same at the start of the study (30 min) but the PNEBoost group did receive two additional 10-min video presentations. These booster sessions fit with current educational studies demonstrating that repeating education provides some advantages in knowledge recall and retention. The results only pertain to six-month follow-up, with no indication how these results transfer to true long-term follow-up, i.e., years later. The six-month behavioral changes were subjective data which are influenced by bias, ability to recall information, etc.

## 5. Conclusions

A PNE session, followed by booster sessions yield more positive behavioral results in middle school children at six-month follow-up than a single PNE session or current school curriculums on pain, including significant reduction in pain medication use. Early pain experiences powerfully influence school, PE, recess and sports attendance/participation, which can be positively influenced by a PNEBoost program.

## Figures and Tables

**Table 1:** Participant Demographics.

	PNE (*n* = 220)	UC (*n* = 198)	PNEBoost (*n* = 250)	Overall (*n* = 668)
Age, mean, years	12.3	12.3	12.2	12.3
Female, *n* (%)	105 (47.7)	96 (48.5)	126 (50.4)	327 (49)
Currently experiencing pain pre-test, *n* (% yes)	84 (38.0)	53 (26.7)	101 (40.4)	238 (35.6)
Mean pain rating of those presenting with pain (NPRS)	1.5	0.8	1.8	1.3
Have experienced pain >3 months, *n* (%)	38 (17.3)	16 (8.1)	42 (16.8)	96 (14.4)
Know someone with chronic pain, *n* (%)	151 (68.6)	149 (75.3)	188 (75.2)	488 (73.1)
Participate in sports, *n* (%)	195 (88.6)	158 (79.8)	205 (82)	558 (83.5)
Mean NPQ score	3.2	3.2	3.1	3.2
Mean FABQ-PA score	12.9	13.5	14.5	13.7
FABQ-PA above cut-off, *n* (%)	93 (42.3)	91 (46)	135 (54)	319 (47.8)

**Table 2:** Descriptive statistics, with and without outliers, for the comparison among the 3 treatment groups for each of the 6-month outcome variables.

		*N*	Mean	SD	SE	95% Confidence Interval		Minimum	Maximum
						Lower	Upper		
School attendance—days missed	PNE	218	0.56	1.21	0.08	0.40	0.73	0	10
	UC	195	0.66	1.84	0.13	0.40	0.92	0	20
	PNEBoost	218	0.85	2.60	0.18	0.51	1.20	0	24
School attendance—days missed (outliers removed)	PNE	216	0.49	0.933	0.06	0.37	0.62	0	5
	UC	193	0.53	1.11	0.08	0.37	0.69	0	6
	PNEBoost	213	0.54	1.18	0.08	0.38	0.69	0	6
Physical education—days missed	PNE	216	0.84	2.61	0.18	0.49	1.19	0	28
	UC	192	0.88	3.10	0.22	0.44	1.32	0	35
	PNEBoost	222	2.34	13.38	0.90	0.57	4.11	0	174
Physical education—days missed (outliers removed)	PNE	215	0.72	1.84	0.13	0.47	0.96	0	15
	UC	191	0.70	1.88	0.14	0.44	0.97	0	16
	PNEBoost	220	1.16	3.11	0.21	0.74	1.57	0	21
Sports participation—days missed	PNE	216	1.06	2.50	0.17	0.73	1.40	0	28
	UC	187	1.36	3.27	0.24	0.89	1.83	0	24
	PNEBoost	220	1.86	7.17	0.48	0.91	2.82	0	90
Sports participation—days missed (outliers removed)	PNE	215	0.94	1.70	0.12	0.71	1.17	0	10
	UC	185	1.11	2.28	0.17	0.78	1.44	0	15
	PNEBoost	213	0.92	2.34	0.16	0.61	1.24	0	14
Recess participation—days missed	PNE	216	0.78	3.04	0.21	0.37	1.19	0	28
	UC	191	1.02	5.24	0.38	0.27	1.77	0	42
	PNEBoost	218	0.41	2.15	0.15	0.13	0.70	0	30
Recess participation—days missed (outliers removed)	PNE	212	0.42	1.22	0.08	0.25	0.58	0	7
	UC	187	0.28	1.13	0.08	0.12	0.45	0	10
	PNEBoost	217	0.28	0.77	0.05	0.17	0.38	0	6
Doctor visits for pain	PNE	208	1.63	4.08	0.28	1.08	2.19	0	50
	UC	178	1.32	2.40	0.18	0.96	1.67	0	19
	PNEBoost	202	1.70	3.28	0.23	1.24	2.15	0	30
Doctor visits for pain (outliers removed)	PE	205	1.28	1.94	0.14	1.01	1.55	0	10
	UC	176	1.14	1.71	0.13	0.88	1.39	0	8
	PNEBoost	198	1.36	2.04	0.15	1.07	1.64	0	10
Rehab visits for pain	PNE	215	1.43	7.86	0.54	0.38	2.49	0	100
	UC	190	0.75	2.68	0.19	0.36	1.13	0	29
	PNEBoost	219	0.71	2.67	0.18	0.35	1.06	0	32
Rehab visits for pain (outliers for pain)	PNE	213	0.73	1.98	0.14	0.47	1.00	0	14
	UC	189	0.60	1.72	0.13	0.35	0.84	0	12
	PNEBoost	218	0.56	1.62	0.11	0.35	0.78	0	10
NPQ 6 month	PNE	220	4.82	2.30	0.16	4.51	5.12	0	12
	UC	194	4.52	2.51	0.18	4.16	4.88	0	12
	PNEBoost	229	5.14	2.10	0.14	4.86	5.41	0	11
FABQ-PA 6 month	PNE	220	1.58	0.50	0.03	1.51	1.64	1	2
	UC	196	1.58	0.50	0.04	1.51	1.65	1	2
	PNEBoost	229	1.60	0.49	0.03	1.53	1.66	1	2

**Table 3:** *p*-Values for the interactions and main effects among group by demographic variables for each of the 6-month outcomes.

	Group X Gender	Group Main Effect	Gender Main Effect
School attendance	*p* = 0.946	*p* = 0.918	*p* = 0.117
PE participation	*p =* 0.269	*p* = 0.092	*p* = 0.001 *
Sports participation	*p* = 0.052	*p* = 0.618	*p* = 0.518
Recess participation	*p* = 0.695	*p* = 0.314	*p* = 0.325
Doctor visits for pain	*p* = 0.607	*p* = 0.534	*p* = 0.398
Rehab visits for pain	*p* = 0.370	*p* = 0.554	*p* = 0.052
rNPQ	*p* = 0.173	*p* = 0.030 *	*p* = 0.667
FABQ-PA	*p* = 0.448	*p* = 0.577	*p* < 0.001 *
	**Group X Pain at Intervention**	**Group Main Effect**	**Pain at Intervention Main Effect**
School attendance	*p* = 0.343	*p* = 0.907	*p* = 0.005 *
PE participation	*p* = 0.358	*p* = 0.177	*p* = 0.070
Sports participation	*p* = 0.393	*p* = 0.313	*p* = 0.115
Recess participation	*p* = 0.466	*p* = 0.156	*p* = 0.221
Doctor visits for pain	*p* = 0.111	*p* = 0.486	*p* = 0.013 *
Rehab visits for pain	*p* = 0.010 *	*p* = 0.198	*p* = 0.074
rNPQ	*p* = 0.509	*p* = 0.025 *	*p* = 0.482
FABQ-PA	*p* = 0.996	*p* = 0.567	*p* = 0.519
	**Group X Chronic Pain at Intervention**	**Group Main Effect**	**Chronic Pain at Intervention Main effect**
School attendance	*p* = 0.004 *	*p* = 0.015 *	*p* < 0.001 *
PE participation	*p* = 0.276	*p* = 0.039 *	*p* < 0.001 *
Sports participation	*p* = 0.611	*p* = 0.290	*p* < 0.001 *
Recess participation	*p* = 0.539	*p* = 0.213	*p* = 0.220
Doctor visits for pain	*p* = 0.332	*p* = 0.829	*p* < 0.001 *
Rehab visits for pain	*p* = 0.082	*p* = 0.088	*p* = 0.014 *
rNPQ	*p* = 0.381	*p* = 0.291	*p* = 0.021 *
FABQ-PA	*p* = 0.616	*p* = 0.976	*p* = 0.052
	**Group X Knowing Someone with Chronic Pain**	**Group Main Effect**	**Knowing Someone with Chronic Pain Main Effect**
School attendance	*p* = 0.501	*p* = 0.970	*p* = 0.001 *
PE participation	*p* = 0.807	*p* = 0.104	*p* = 0.045 *
Sports participation	*p* = 0.633	*p* = 0.972	*p* = 0.007 *
Recess participation	*p* = 0.212	*p* = 0.096	*p* = 0.641
Doctor visits for pain	*p* = 0.503	*p* = 0.670	*p* = 0.006 *
Rehab visits for pain	*p* = 0.053	*p* = 0.739	*p* = 0.048 *
rNPQ	*p* = 0.010 *	*p* = 0.531	*p* = 0.051
FABQ-PA	*p* < 0.001 *	*p* = 0.036 *	*p* = 0.166
	**Group X Fear of Physical Activity at Time of Intervention**	**Group Main Effect**	**Fear of Physical Activity at Time of Intervention Main Effect**
School attendance	*p* = 0.779	*p* = 0.892	*p* = 0.541
PE participation	*p* = 0.330	*p* = 0.108	*p* = 0.002 *
Sports participation	*p* = 0.658	*p* = 0.642	*p* = 0.002 *
Recess participation	*p* = 0.374	*p* = 0.409	*p* = 0.079
Doctor visits for pain	*p* = 0.816	*p* = 0.534	*p* = 0.361
Rehab visits for pain	*p* = 0.708	*p* = 0.602	*p* = 0.777
rNPQ	*p* = 0.923	*p* = 0.015 *	*p* = 0.657
FABQ-PA	*p* = 0.085	*p* = 0.423	*p* < 0.001 *
	**Group X Pain Knowledge Change from Intervention**	**Group Main Effect**	**Pain Knowledge Change from Intervention Main Effect**
School attendance	*p* = 0.023 *	*p* = 0.538	*p* = 0.727
PE participation	*p* = 0.705	*p* = 0.147	*p* = 0.793
Sports participation	*p* = 0.296	*p* = 0.313	*p* = 0.430
Recess participation	*p* = 0.223	*p* = 0.186	*p* = 0.004 *
Doctor visits for pain	*p* = 0.157	*p* = 0.492	*p* = 0.114
Rehab visits for pain	*p* = 0.072	*p* = 0.275	*p* = 0.604
rNPQ	*p* = 0.049 *	*p* = 0.264	*p* = 0.373
FABQ-PA	*p* = 0.735	*p* = 0.806	*p* = 0.887

All statistically significant results are highlighted with an asterisk (*).

**Table 4:** Detailed findings for each of the regression analyses.

		Unstandardized Coefficients		Standardized Coefficients	t	*p* Value
		B	Standard Error	Beta		
School attendance *p* < 0.001 R = 0.252 R^2^ = 0.064	(Constant)	−0.207	0.206		−1.006	0.315
	Gender	0.187	0.102	0.081	1.830	0.068
	Duration	0.242	0.068	0.159	3.567	<0.001 *
	Knowing someone	0.265	0.125	0.094	2.129	0.034 *
	rNPQ improvement	0.050	0.020	−0.113	−2.557	0.011 *
	FABQ change	0.002	0.009	0.010	0.218	0.828
	Pain rating	0.035	0.024	0.065	1.455	0.146
PE participation *p* < 0.001 R = 0.261 R^2^ = 0.068	(Constant)	−1.119	0.459		−2.438	0.015
	Gender	0.834	0.227	0.161	3.666	<0.001 *
	Duration	0.546	0.152	0.159	3.579	<0.001 *
	Knowing someone	0.118	0.279	0.019	0.422	0.674
	rNPQ improvement	0.012	0.043	−0.012	−0.265	0.791
	FABQ change	0.053	0.021	0.112	2.539	0.011 *
	Pain rating	0.004	0.053	0.004	0.083	0.934
Sports participation *p* < 0.001 R = 0.243 R^2^=0.059	(Constant)	−0.262	0.394		−0.666	0.506
	Gender	0.161	0.194	0.037	0.829	0.408
	Duration	0.558	0.130	0.194	4.283	<0.001 *
	Knowing someone	0.403	0.237	0.076	1.702	0.089
	rNPQ improvement	0.012	0.037	−0.014	−0.313	0.754
	FABQ change	0.034	0.018	0.085	1.887	0.060
	Pain rating	−0.020	0.045	−0.020	−0.433	0.665
Recess participation *p* = 0.033 R = 0.165 R^2^ = 0.027	(Constant)	0.401	0.206		1.944	0.052
	Gender	−0.042	0.102	−0.019	−0.416	0.678
	Duration	0.156	0.068	0.105	2.303	0.022 *
	Knowing someone	0.145	0.124	0.053	−1.162	0.246
	rNPQ improvement	0.042	0.020	−0.097	−2.143	0.033 *
	FABQ change	0.014	0.009	0.067	1.482	0.139
	Pain rating	0.015	0.024	0.029	0.626	0.532

All statistically significant results are highlighted with an asterisk (*).

**Table 5:** Factor predictive of school attendance, physical education (PE) attendance, recess attendance and sports participation 6 months later.

	Having Experienced Pain >3 Months	Knowledge of Pain (rNPQ)	Knowing Someone with Chronic Pain	Gender	High Fear of Physical Activity (FABQ-PA)
School attendance	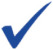	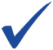	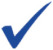		
PE attendance	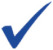			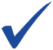	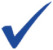
Sports participation	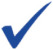				
Recess attendance	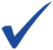	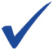			

## Appendix A

**Table A1:** Descriptive statistics for Aim 2.

School Attendance					PE Participation				
Group	Gender	Mean	SD	*N*	Group	Gender	Mean	SD	*N*
PNE	Boy	0.41	0.82	112	PNE	Boy	0.56	1.70	111
	Girl	0.58	1.04	104		Girl	0.88	1.96	104
UC	Boy	0.48	0.98	100	UC	Boy	0.44	0.99	97
	Girl	0.59	1.24	93		Girl	0.97	2.46	94
PNEBoost	Boy	0.47	1.11	103	PNEBoost	Boy	0.63	1.89	105
	Girl	0.59	1.26	106		Girl	1.67	3.91	111
**Sports participation**					**Recess participation**				
**Group**	**Gender**	**Mean**	**SD**	***N***	**Group**	**Gender**	**Mean**	**SD**	***N***
PNE	Boy	1.11	1.90	112	PNE	Boy	0.45	1.28	111
	Girl	0.76	1.44	103		Girl	0.38	1.14	101
UC	Boy	1.09	2.10	95	UC	Boy	0.37	1.17	94
	Girl	1.13	2.46	90		Girl	0.19	1.09	93
PNEBoost	Boy	0.61	1.28	102	PNEBoost	Boy	0.27	0.85	103
	Girl	1.25	3.02	107		Girl	0.27	0.68	110
**Visits to the doctor for pain**					**Visits to rehabilitation for pain**				
**Group**	**Gender**	**Mean**	**SD**	***N***	**Group**	**Gender**	**Mean**	**SD**	***N***
PNE	Boy	1.13	1.85	103	PNE	Boy	0.58	1.82	111
	Girl	1.43	2.03	102		Girl	0.09	2.13	102
UC	Boy	1.18	1.82	89	UC	Boy	0.59	1.78	96
	Girl	1.10	1.60	87		Girl	0.60	1.66	93
PNEBoost	Boy	1.27	2.26	93	PNEBoost	Boy	0.31	1.05	101
	Girl	1.45	1.84	102		Girl	0.81	2.00	113
**rNPQ**					**FABQ**				
**Group**	**Gender**	**Mean**	**SD**	***N***	**Group**	**Gender**	**Mean**	**SD**	***N***
PNE	Boy	5.06	2.36	115	PNE	Boy	13.50	5.27	115
	Girl	4.55	2.20	105		Girl	14.60	4.91	105
UC	Boy	4.54	2.39	99	UC	Boy	13.42	5.59	100
	Girl	4.51	2.65	95		Girl	15.68	4.60	96
PNEBoost	Boy	4.96	2.11	109	PNEBoost	Boy	13.55	4.52	109
	Girl	5.27	2.06	116		Girl	14.90	4.24	116
**School attendance**					**PE participation**				
**Group**	**Gender**	**Mean**	**SD**	***N***	**Group**	**Gender**	**Mean**	**SD**	***N***
PNE	No	0.39	0.84	131	PNE	No	0.61	1.96	131
	Yes	0.65	1.06	83		Yes	0.89	1.63	82
UC	No	0.51	1.12	141	UC	No	0.49	1.08	137
	Yes	0.60	1.11	51		Yes	1.26	3.06	53
PNEBoost	No	0.35	1.00	119	PNEBoost	No	1.15	3.48	125
	Yes	0.78	1.36	90		Yes	1.20	2.61	91
**Sports participation**					**Recess participation**				
**Group**	**Gender**	**Mean**	**SD**	***N***	**Group**	**Gender**	**Mean**	**SD**	***N***
PNE	No	0.78	1.60	131	PNE	No	0.32	1.07	129
	Yes	1.11	1.74	82		Yes	0.58	1.42	81
UC	No	0.96	2.19	133	UC	No	0.27	1.14	135
	Yes	1.53	2.47	51		Yes	0.27	1.10	51
PNEBoost	No	0.95	2.65	120	PNEBoost	No	0.23	0.70	120
	Yes	0.91	1.90	89		Yes	0.30	0.83	93
**Visits to the doctor for pain**					**Visits to rehabilitation for pain**				
**Group**	**Gender**	**Mean**	**SD**	***N***	**Group**	**Gender**	**Mean**	**SD**	***N***
PNE	No	0.91	1.57	124	PNE	No	0.42	1.45	129
	Yes	1.81	2.32	79		Yes	1.24	2.55	82
UC	No	1.09	1.64	125	UC	No	0.54	1.39	136
	Yes	1.20	1.85	50		Yes	0.75	2.39	52
PNEBoost	No	1.25	2.14	110	PNEBoost	No	0.63	1.82	123
	Yes	1.50	1.94	84		Yes	0.41	1.11	91
**rNPQ**					**FABQ**				
**Group**	**Gender**	**Mean**	**SD**	***N***	**Group**	**Gender**	**Mean**	**SD**	***N***
PNE	No	4.79	2.32	134	PNE	No	13.93	5.17	134
	Yes	4.90	2.28	84		Yes	14.14	5.11	84
UC	No	4.56	2.62	140	UC	No	14.45	5.57	142
	Yes	4.43	2.23	53		Yes	14.74	4.35	53
PNEBoost	No	4.95	2.19	131	PNEBoost	No	14.09	4.52	131
	Yes	5.37	2.00	94		Yes	14.39	4.29	94
**School attendance**					**PE participation**				
**Group**	**Chronic pain**	**Mean**	**SD**	***N***	**Group**	**Chronic pain**	**Mean**	**SD**	***N***
PNE	No	0.43	0.85	180	PNE	No	0.62	1.69	180
	Yes	0.81	1.24	36		Yes	1.20	2.45	35
UC	No	0.43	0.93	177	UC	No	0.59	1.53	174
	Yes	1.63	2.09	16		Yes	1.82	3.88	17
PNEBoost	No	0.53	1.19	177	PNEBoost	No	0.92	2.58	186
	Yes	0.58	1.11	36		Yes	2.47	4.97	34
**Sports participation**					**Recess participation**				
**Group**	**Chronic pain**	**Mean**	**SD**	***N***	**Group**	**Chronic pain**	**Mean**	**SD**	***N***
PNE	No	0.83	1.56	178	PNE	No	0.37	1.22	177
	Yes	1.49	2.21	37		Yes	0.66	1.19	35
UC	No	1.01	2.20	170	UC	No	0.27	1.09	172
	Yes	2.33	2.80	15		Yes	0.47	1.55	15
PNEBoost	No	0.81	2.22	181	PNEBoost	No	0.28	0.70	180
	Yes	1.59	2.86	32		Yes	0.27	1.05	37
					Total	No	0.30	1.02	529
						Yes	0.46	1.20	87
**Recess participation**					**Visits to the doctor for pain**				
**Group**	**Chronic pain**	**Mean**	**SD**	***N***	**Group**	**Chronic pain**	**Mean**	**SD**	***N***
PNE	No	0.37	1.22	177	PNE	No	1.05	1.65	168
	Yes	0.66	1.19	35		Yes	2.32	2.70	37
UC	No	0.27	1.09	172	UC	No	1.00	1.58	160
	Yes	0.47	1.55	15		Yes	2.56	2.28	16
PNEBoost	No	0.28	0.70	180	PNEBoost	No	1.23	1.95	163
	Yes	0.27	1.05	37		Yes	1.97	2.33	35
Total	No	0.30	1.02	529					
	Yes	0.46	1.20	87					
**Visits to rehabilitation for pain**					**rNPQ**				
**Group**	**Chronic pain**	**Mean**	**SD**	***N***	**Group**	**Chronic pain**	**Mean**	**SD**	***N***
PNE	No	0.54	1.75	177	PNE	No	4.82	2.30	182
	Yes	1.69	2.68	36		Yes	4.82	2.34	38
UC	No	0.58	1.75	173	UC	No	4.60	2.51	177
	Yes	0.81	1.38	16		Yes	3.71	2.42	17
PNEBoost	No	0.53	1.55	183	PNEBoost	No	5.13	2.13	191
	Yes	0.74	1.96	35		Yes	5.16	2.01	38
**FABQ**					**School attendance**				
**Group**	**Chronic pain**	**Mean**	**SD**	***N***	**Group**	**Know someone**	**Mean**	**SD**	***N***
PNE	No	14.09	5.11	182	PNE	No	0.36	0.69	68
	Yes	13.71	5.22	38		Yes	0.54	1.02	147
UC	No	14.66	5.30	179	UC	No	0.21	0.45	46
	Yes	13.06	4.49	17		Yes	0.65	1.24	144
PNEBoost	No	14.48	4.28	191	PNEBoost	No	0.23	0.59	48
	Yes	13.05	4.83	38		Yes	0.62	1.29	161
**PE participation**					**Sports participation**				
**Group**	**Know someone**	**Mean**	**SD**	***N***	**Group**	**Know someone**	**Mean**	**SD**	***N***
PNE	No	0.31	0.93	67	PNE	No	0.61	1.23	67
	Yes	0.89	2.17	147		Yes	1.05	1.82	147
UC	No	0.34	0.80	46	UC	No	0.49	1.25	45
	Yes	0.84	2.12	142		Yes	1.29	2.47	138
PNEBoost	No	0.97	2.63	50	PNEBoost	No	0.67	2.16	49
	Yes	1.22	3.27	166		Yes	1.03	2.41	160
	Total	1.16	3.13	216					
**Recess participation**					**Visits to the doctor for pain**				
**Group**	**Know someone**	**Mean**	**SD**	***N***	**Group**	**Know someone**	**Mean**	**SD**	***N***
PNE	No	0.54	1.35	67	PNE	No	0.72	1.29	65
	Yes	0.36	1.15	144		Yes	1.50	2.10	139
UC	No	0.22	0.94	46	UC	No	0.86	1.39	42
	Yes	0.31	1.20	139		Yes	1.19	1.78	132
PNEBoost	No	0.10	0.37	49	PNEBoost	No	1.04	1.96	47
	Yes	0.32	0.84	164		Yes	1.41	2.05	147
**Visits to rehabilitation for pain**					**rNPQ**				
**Group**	**Know someone**	**Mean**	**SD**	***N***	**Group**	**Know someone**	**Mean**	**SD**	***N***
PNE	No	0.24	0.75	66	PNE	No	4.82	2.53	68
	Yes	0.94	2.30	146		Yes	4.83	2.19	151
UC	No	0.22	0.88	45	UC	No	5.56	3.11	45
	Yes	0.73	1.91	142		Yes	4.21	2.24	146
PNEBoost	No	0.74	1.89	50	PNEBoost	No	5.04	1.83	51
	Yes	0.52	1.55	165		Yes	5.15	2.19	174
**FABQ**					**School attendance**				
**Group**	**Know someone**	**Mean**	**SD**	***N***	**Group**	**FABQ group**	**Mean**	**SD**	***N***
PNE	No	12.07	5.85	68	PNE	No	0.48	0.93	70
	Yes	14.99	4.40	151		Yes	0.51	0.95	141
UC	No	15.63	5.87	46	UC	No	0.57	1.21	71
	Yes	14.21	4.97	147		Yes	0.50	1.07	117
PNEBoost	No	13.96	4.39	51	PNEBoost	No	0.60	1.29	91
	Yes	14.30	4.37	174		Yes	0.48	1.09	121
**PE participation**					**Sports participation**				
**Group**	**FABQ group**	**Mean**	**SD**	***N***	**Group**	**FABQ group**	**Mean**	**SD**	***N***
PNE	No	1.28	2.77	69	PNE	No	1.37	2.10	67
	Yes	0.46	1.10	142		Yes	0.73	1.45	143
UC	No	0.85	2.17	71	UC	No	1.35	2.70	66
	Yes	0.64	1.72	115		Yes	1.03	2.04	114
PNEBoost	No	1.66	4.06	92	PNEBoost	No	1.33	2.96	90
	Yes	0.79	2.13	127		Yes	0.63	1.70	122
**Recess participation**					**Visits to the doctor for pain**				
**Group**	**FABQ group**	**Mean**	**SD**	***N***	**Group**	**FABQ group**	**Mean**	**SD**	***N***
PNE	No	0.43	1.40	65	PNE	No	1.45	2.05	65
	Yes	0.42	1.14	142		Yes	1.21	1.91	135
UC	No	0.39	1.51	67	UC	No	1.15	1.71	64
	Yes	0.23	0.87	116		Yes	1.15	1.74	107
PNEBoost	No	0.45	1.02	92	PNEBoost	No	1.48	2.20	81
	Yes	0.14	0.45	124		Yes	1.26	1.93	116
**Visits to rehabilitation for pain**					**rNPQ**				
**Group**	**FABQ group**	**Mean**	**SD**	***N***	**Group**	**FABQ group**	**Mean**	**SD**	***N***
PNE	No	0.75	1.98	68	PNE	No	4.70	2.22	71
	Yes	0.71	1.98	140		Yes	4.81	2.34	144
UC	No	0.70	1.93	66	UC	No	4.39	2.43	71
	Yes	0.50	1.48	118		Yes	4.56	2.49	118
PNEBoost	No	0.51	1.77	91	PNEBoost	No	5.14	2.07	97
	Yes	0.61	1.52	126		Yes	5.13	2.14	131
**FABQ**					**School attendance**				
**Group**	**FABQ group**	**Mean**	**SD**	***N***	**Group**	**rNPQ improvement**	**Mean**	**SD**	***N***
PNE	No	16.87	4.50	71	PNE	No	0.47	0.96	136
	Yes	12.72	4.88	144		Yes	0.53	0.89	80
UC	No	16.65	4.68	72	UC	No	0.79	1.41	38
	Yes	13.32	5.21	119		Yes	0.47	1.02	155
PNEBoost	No	15.45	3.91	97	PNEBoost	No	0.40	0.96	136
	Yes	13.33	4.54	131		Yes	0.76	1.47	76
**PE participation**					**Sports participation**				
**Group**	**rNPQ improvement**	**Mean**	**SD**	***N***	**Group**	**rNPQ improvement**	**Mean**	**SD**	***N***
PNE	No	0.63	1.91	136	PNE	No	0.85	1.51	135
	Yes	0.87	1.71	79		Yes	1.09	1.98	80
UC	No	0.87	2.52	38	UC	No	1.53	2.60	36
	Yes	0.66	1.69	153		Yes	1.01	2.19	149
PNEBoost	No	1.11	3.07	144	PNEBoost	No	0.99	2.43	137
	Yes	1.24	3.21	75		Yes	0.81	2.18	75
**Recess participation**					**Visits to the doctor for pain**				
**Group**	**rNPQ improvement**	**Mean**	**SD**	***N***	**Group**	**rNPQ improvement**	**Mean**	**SD**	***N***
PNE	No	0.26	0.87	135	PNE	No	1.17	1.84	130
	Yes	0.69	1.62	77		Yes	1.47	2.11	75
UC	No	0.26	0.69	38	UC	No	1.27	1.99	33
	Yes	0.29	1.22	149		Yes	1.11	1.65	143
PNEBoost	No	0.14	0.47	140	PNEBoost	No	1.10	1.67	129
	Yes	0.50	1.08	76		Yes	1.82	2.56	68
**Visits to rehabilitation for pain**					**rNPQ**				
**Group**	**rNPQ improvement**	**Mean**	**SD**	***N***	**Group**	**rNPQ improvement**	**Mean**	**SD**	***N***
PNE	No	0.60	1.63	134	PNE	No	4.66	2.39	138
	Yes	0.96	2.45	79		Yes	5.09	2.13	82
UC	No	0.73	1.47	37	UC	No	4.76	2.57	38
	Yes	0.57	1.78	152		Yes	4.46	2.50	156
PNEBoost	No	0.72	1.92	145	PNEBoost	No	5.37	2.14	147
	Yes	0.26	0.65	72		Yes	4.70	1.99	81
**FABQ**									
**Group**	**rNPQ improvement**	**Mean**	**SD**	***N***					
PNE	No	13.93	5.02	138					
	Yes	14.18	5.32	82					
UC	No	14.26	5.36	39					
	Yes	14.59	5.23	157					
PNEBoost	No	14.37	4.05	147					
	Yes	13.98	4.98	81					
